# Adherence to Mediterranean diet, physical activity level, and severity of periodontitis: Results from a university‐based cross‐sectional study

**DOI:** 10.1002/JPER.21-0643

**Published:** 2022-02-25

**Authors:** Crystal Marruganti, Jacopo Traversi, Carlo Gaeta, Edoardo Ferrari Cagidiaco, Stefano Parrini, Nicola Discepoli, Simone Grandini

**Affiliations:** ^1^ Unit of Periodontology, Endodontology and Restorative Dentistry Department of Medical Biotechnologies University of Siena Siena Italy; ^2^ Unit of Prosthodontics Department of Medical Biotechnologies University of Siena Siena Italy; ^3^ Unit of Oral Surgery Department of Medical Biotechnologies University of Siena Siena Italy

**Keywords:** chronic periodontitis, Mediterranean diet, periodontal attachment loss, physical activity

## Abstract

**Background:**

The aim of this study was to evaluate the association between adherence to Mediterranean diet (MD) and physical activity (PA) level with the periodontal status of a University‐based cohort of individuals.

**Methods:**

A total of 235 individuals were included in the study. MD adherence and PA level were registered through validated questionnaires, together with a full periodontal examination. Crude and adjusted odds ratios (ORs) [95% confidence interval] were calculated to evaluate the association between MD adherence, PA level, and periodontitis severity. A final logistic multivariate regression model was built to evaluate the impact of the combination between low MD adherence and low PA level on the prevalence of Stage III/IV periodontitis.

**Results:**

The adjusted ORs for Stage III/IV periodontitis were 1.65 [0.84 to 3.28; *P *= 0.42] for low PA and 5.63 [3.21 to 9.84; *P *= 0.00] for low MD adherence. The final logistic multivariate regression model resulted in OR = 10.23 [4.01, 26.09; *P *= 0.00] of having Stage III/IV periodontitis in individuals with low MD adherence and low PA.

**Conclusions:**

Individuals conducting a lifestyle characterized by the combination of low MD adherence and lack of regular exercise had 10 times the odds to have severe forms of periodontitis.

## 1 INTRODUCTION

Periodontitis is defined as a biofilm‐mediated non‐communicable chronic inflammatory disease (NCD) characterized by the progressive destruction of the tooth supporting apparatus. Periodontitis is a highly common chronic inflammatory NCD, with a prevalence of its severe form between 7% and 11%;^1^ it represents the sixth most prevalent condition worldwide.^1^ Further, periodontitis is associated with a range of systemic diseases, including diabetes,^2^ cardiovascular disease (CVD),^3^ and adverse pregnancy outcomes.^3^ Several factors, such as overweight, smoking, unhealthy diet, and physical inactivity,^4^ are associated with disease occurrence and are shared as risk indicators with other prevalent NCDs, such as type‐2 diabetes mellitus (T2DM) and CVD.

Indeed, periodontitis exerts a detrimental impact on both masticatory function and general health, thus resulting in higher dental healthcare costs.^5^ Consequently, a lot of research^6^, ^7^, ^8^, ^9^ focused on the treatment of NCDs through a multifactorial approach targeted at improving various aspects of the patient's lifestyle, such as smoking, diet or physical activity (PA). In particular, the pioneer Seven Countries study in the 1950s was the first one to highlight the benefic effects of the Mediterranean diet (MD)^10^; since then, epidemiological evidence flourished regarding the ability of MD to significantly reduce the risk of developing NCDs such as metabolic syndrome, T2DM, CVD, and cancers.^11^, ^12^ The latest consensus on the Mediterranean pyramid^13^ encompassed not only the consumption and serving size of specific food groups, but also other lifestyle dimensions (i.e., regular exercise and adequate rest) which, collectively, were framed in the “Mediterranean lifestyle” (ML).^13^

While on the one hand a lot of studies focused on the effects of each single item of the ML (e.g., PA or nutrition),^9^, ^14^, ^15^ no data are present regarding the impact of the combination of ML components or ML as a whole on oral health. In particular, while there is accumulating evidence^16^, ^17^, ^18^ regarding the anti‐inflammatory potential of MD, on the other hand no evidence is present regarding the synergistic/antagonistic impact of adherence to both MD and PA on periodontal health. The hypothesis that we would like to figure out with this current cross‐sectional design is that low MD adherence combined with a sedentary behavior would increase low‐grade systemic inflammation, lipid levels and oxidative stress, as well as decrease insulin sensitivity^19^, ^20^, ^21^; as such, they would contribute to a more severe periodontitis phenotype. Therefore, the aim of the present cross‐sectional study was to evaluate the association between MD adherence and PA level with the biometric and inflammatory periodontal parameters of a University‐based cohort of individuals.

## 2 MATERIALS AND METHODS

### 2.1 Study design

The present study is reported according to the Strengthening the Reporting of Observational studies in Epidemiology (STROBE) guidelines for cross‐sectional studies.^22^ The research protocol was approved by the local ethics committee (protocol number: 18993/2021) and received the registration number on Clinicaltrials.gov (NCT04771949).

### 2.2 Setting and participants

All consecutive patients attending the Unit of Periodontology at the University of Siena were screened between January 2021 and August 2021; the inclusion criteria were:

‐ age between 18 and 70 years old;
‐ ability and willingness to give informed consent.
The exclusion criteria were:
‐ pregnancy or lactation;
‐ periodontal therapy performed in the last 12 months;
‐ administration of antibiotics within the last 6 months;
‐ inability to communicate effectively in Italian.

Individuals were included in the study after they read and signed the written informed consent, in accordance with the Declaration of Helsinki.

### 2.3 Variables

#### 2.3.1 Sociodemographic characteristics

Information regarding patients' age, sex, smoking and oral hygiene habits, occupation and education level was registered. Moreover, data regarding the presence of familiarity as well as any comorbidity affecting susceptibility to periodontitis were recorded. The body mass index (BMI) was computed as weight (kg)/height (m2). The assessment methods of socio‐demographic characteristics are reported in the Supplementary Appendix in the online *Journal of Periodontology*.

#### 2.3.2 Dietary assessment

A validated 15‐item questionnaire to measure patients’ adherence to MD (QueMD) was administered by two examiners (C.M., J.T.), following the structured questions and explanations provided by the questionnaire.^23^ The questionnaire included questions regarding the foods most frequently associated with MD (wholegrain pasta, bread or substitutes; raw or cooked vegetables; all types of fresh fruits; dairies; either red or white wine; olive oil; red meat; fish; dried fruits; and pulses), as well as other commonly consumed items (white meat, carbonated beverages or soft drinks; butter, cooking cream or margarine; manufactured sweets, or pastries) (see Supplementary Table S1 in online *Journal of Periodontology*). For each component, a standard portion for the Italian population was indicated^24^ and participants could choose among five consumption frequencies, which differed according to food items. The alternate MD score (aMed) was drawn from the QueMD results in order to evaluate patients’ adherence to MD^23^; it was calculated by assigning 1 point to participants reporting food consumptions above the Italian National levels^25^ for each of the following items typical of the MD: wholegrain products (≥1/d), vegetables (≥2/d), fresh fruits (≥2/d), olive oil (≥3/d), wine (1 to 2 glasses/d for males or 1 glass/d for females), red meat (≤1 to 3/wk), fish (≥2/wk), dried fruits (≥2/wk), pulses (≥2/d).^13^ The sum score ranged between 0 (minimum MD adherence) and 9 (maximum MD adherence); it was dichotomized in order to define cases of low (aMed <5) and high (aMed >4) adherence to MD considering its median in the current study population as the cut‐off value.

#### 2.3.3 Physical activity assessment

PA was assessed through the validated short version of the International Physical Activity Questionnaire (IPAQ).^26^ It was administered by two examiners (C.M., J.T.), who asked the structured questions and gave the explanations provided by the questionnaire.^26^ It consists of seven items regarding the frequency and amount of time spent doing intense and moderate PA, as well as walking or doing sedentary activities during the last 7 days (Supplementary Table S1). The overall PA level was classified as low, moderate or high through the IPAQ automatic report (https://theipaq/home).

#### 2.3.4 Periodontal examination

All participants received a full periodontal chart by two trained and calibrated examiners (C.M., J.T.) (unweighted *kappa* score of 0.98). Examiners were calibrated by performing a full periodontal chart on two non‐study subjects affected by periodontitis; the examiner was considered reproducible if an agreement of at least 95% of clinical attachment level (CAL) (with maximum a 2 mm difference) between two repeated measurements was recorded. Periodontal probing depth (PPD), gingival recession (REC), plaque^27^ and Bleeding on Probing (BoP)^28^ were recorded with a standardized periodontal probe* six sites per tooth, third molars excluded. Whenever the Cementum Enamel Junction (CEJ) was subgingival, CAL was measured as the difference between PPD and the distance between the free gingival margin and the CEJ. The presence of furcation involvement was recorded according to the classification of Hamp^29^; the classification of Miller^30^ was used to record tooth mobility.

A periodontitis case was defined whenever interdental CAL was detectable at ≥2 nonadjacent teeth, or whenever buccal or oral CAL ≥3 mm with pocketing (PPD >3 mm) was detectable at ≥2 teeth.^31^ Periodontitis severity, complexity of management and extent of distribution were assessed with the Staging^32^; the Grading was used in order to assess the rate of disease progression.^32^ Whenever possible, the Grade was assigned with direct evidence of disease progression (i.e., longitudinal data of radiographic bone loss or CAL over 5 years); whenever these data were not available, then indirect evidence was used (case presentation or % bone loss/age). As for the Grade modifiers, smoking status was self‐reported, while the diagnosis of diabetes was ascertained by checking the patient's medical report; glycohemoglobin levels were taken into account only when available.^32^

### 2.4 Statistical analysis

#### 2.4.1 Sample size calculation

Sample size was calculated considering the prevalence of periodontitis in the reference cohort at 37.3%^33^ and its value in the study cohort as 10% higher. Considering α = 0.05 and β = 0.80, the computed sample size was of 185 subjects. Given a nonresponse rate of 20%, the inclusion of 235 participants was planned.

#### 2.4.2 Descriptive and inferential statistics

Statistical analysis was performed through an ad hoc software† setting the level of significance at α = 0.05. Continuous variables were reported as Mean with 95% Confidence Interval; binomial and categorical data were expressed as number of observations (proportion). After verification of data distribution, Kruskal–Wallis and Fisher's exact tests were used to compare patients’ characteristics according to oral health status, MD adherence, PA level and their possible combination (low aMed and low/moderate PA, low aMed and high PA, high aMed and low/moderate PA, and high aMed and high PA).

#### 2.4.3 Logistic regression models

Univariate and multivariate logistic regression analyses were performed to compute the association between Stage III/IV periodontitis according to PA level, aMed and each component of the aMed score; it was expressed as crude and adjusted odds ratios (ORs). ORs were adjusted for parameters that could affect periodontitis phenotype (i.e., age, sex, smoking, and brushing frequency); these parameters were selected according to external knowledge. A multivariate logistic regression model was then built to evaluate the impact of the combination between MD adherence and PA level (independent variable) on the occurrence of Stage III/IV periodontitis cases (dependent variable). The best model was chosen according to the highest value of AUC, and the lowest values of Akaike (AIC) and Bayesian (BIC) information criteria. The predictors included in the final model encompassed: i) age; ii) smoking; iii) familiarity for periodontitis; iv) presence comorbidities. Moreover, the mediating effect of BMI on the impact of the combination between MD adherence and PA level on the presence of Stage III to IV periodontitis was investigated.

## 3 RESULTS

### 3.1 Participant characteristics

A total of 235 participants were included in the present study. The posthoc power analysis for each outcome, that is, association between Stage III/IV periodontitis and low MD adherence, low PA level and their combination, respectively, is reported in Supplementary Table S2 in the online *Journal of Periodontology*. All individuals examined for eligibility accepted to participate, were enrolled in the study and then included in the analysis. Subjects’ characteristics overall and by periodontal status are reported in Table 1. The mean age was 53.90 [52.01, 55.79] years, with a proportion of 57.87% females and 25.96% smokers; the mean BMI was 25.49 [24.88, 26.11]. Around 20% of subjects were affected by at least one comorbidity. Moreover, half the participants were affected by Stage III/IV periodontitis; significant differences as to age, occupation, education and familiarity for periodontitis were found across subgroups of periodontitis severity (Table 1). No significant differences were reported as to domiciliary oral hygiene procedures.

**TABLE 1:** Descriptive statistics overall and by oral health status

	TOTAL	Healthy‐gingivitis	Stage I periodontitis	Stage II periodontitis	Stage III/IV periodontitis	*P*‐value*
Variable	n = 235	n = 34	n = 27	n = 56	n = 118	
*Sociodemographic characteristics*						
Age	53.90 [52.01, 55.79]	38.81^a^ [32.78, 44.85]	48.29^a,c^ [42.48, 54.09]	53.86^b,c^ [50.01, 57.71]	58.49^d^ [56.76, 61.34]	*0.00*
BMI	25.49 [24.88, 26.11]	23.79^a^ [22.44, 25.12]	24.37^a^ [22.73, 26.01]	23.94^a^ [22.91, 24.97]	26.98^b^ [26.04, 27.91]	*0.00*
Sex, females	136 (57.87%)	20 (58.82%)	17 (62.96%)	38 (67.86%)	61 (51.69%)	0.29
Occupation						
Unemployed	42 (17.95%)	9^a^ (27.27%)	7^a,b^ (25.93%)	3^b^ (5.36%)	23^a^ (19.49%)	*0.04*
Employed	133 (56.84%)	20^a^ (60.61%)	16^a,b^ (59.26%)	39^b^ (69.64%)	58^a^ (49.15%)	
Retired	59 (25.21%)	4^a^ (12.12%)	4^a,b^ (14.81%)	14^b^ (25%)	37^a^ (31.36%)	
Education						*0.00*
Elementary/middle school	69 (29.49%)	6^a^ (17.65%)	1^a^ (3.70%)	12^a^ (21.82%)	50^b^ (42.37%)	
High school	107 (45.73%)	18^a^ (52.94%)	14^a^ (51.85%)	29^a^ (52.73%)	46^b^ (38.98%)	
College or more	58 (24.79%)	10^a^ (29.41%)	12^a^ (44.44%)	14^a^ (25.45%)	22^b^ (18.64%)	
Smoking						0.12
Never	107 (45.53%)	22 (64.71%)	15 (55.56%)	26 (46.43%)	44 (37.29%)	
Former	67 (28.51%)	5 (14.71%)	4 (14.81%)	19 (33.93%)	39 (33.05%)	
Smoker	61 (25.96%)	7 (20.59%)	8 (29.63%)	11 (19.64%)	35 (29.66%)	
Familiarity for periodontitis, yes	87 (37.02%)	8 (23.53%)	8 (29.63%)	19 (33.93%)	52 (44.07%)	0.06
Diabetes						0.75
No	180 (76.60%)	24 (70.59%)	21 (77.78%)	43 (76.79%)	91 (77.12%)	
Familiarity	44 (18.72%)	8 (23.53%)	4 (14.81%)	12 (21.43%)	20 (16.95%)	
Yes	11 (4.68%)	2 (5.88%)	2 (7.41%)	1 (1.79%)	7 (5.93%)	
Rheumatoid arthritis						0.82
No	198 (84.26%)	28 (82.35%)	24 (88.89%)	48 (85.71%)	98 (83.05%)	
Familiarity	18 (7.66%)	4 (11.76%)	1 (3.70%)	5 (8.93%)	8 (6.78%)	
Yes	19 (8.09%)	2 (5.88%)	2 (7.41%)	3 (5.36%)	12 (10.17%)	
Inflammatory bowel diseases					0.87	
No	224 (95.32%)	31 (91.18%)	26 (96.30%)	54 (96.43%)	113 (95.76%)	
Familiarity	6 (2.55%)	2 (5.88%)	0 (0%)	1 (1.79%)	3 (2.54%)	
Yes	5 (2.13%)	1 (2.94%)	1 (3.70%)	1 (1.79%)	2 (1.69%)	
Osteoporosis						0.22
No	192 (81.70%)	31 (91.18%)	24 (88.89%)	44 (78.57%)	93 (78.81%)	
Familiarity	29 (12.34%)	3 (8.82%)	3 (11.11%)	10 (17.86%)	13 (11.02%)	
Yes	14 (5.96%)	0 (0%)	0 (0%)	2 (3.57%)	12 (10.17%)	
aMed score^†^	4.31 [4.05, 4.56]	5.35^a^ [3.61, 6.09]	6.07^a^ [5.55, 6.59]	5.13^a^ [4.71, 5.54]	3.5^c^ [3.16, 3.84]	*0.00*
High aMed	118 (50.42%)	17^a^ (50%)	26^a^ (96.30%)	40^b^ (71.43%)	35^c^ (29.66%)	*0.00*
Low aMed	117 (49.58%)	17^a^ (50%)	1^a^ (3.70%)	16^b^ (28.57%)	83^c^ (70.34%)	
PA level						*0.04*
High PA	67 (28.81%)	10^a^ (29.41%)	12^a^ (44.44%)	19^a^ (33.93%)	26^b^ (22.03%)	
Low/moderate PA	168 (71.19%)	24^a^ (70.59%)	15^a^ (55.56%)	37^a^ (66.07%)	92^b^ (77.97%)	
*Oral health status*						
Number of teeth	24.17 [23.47, 24.86]	27.14^a^ [25.93, 28.36]	25.33^a^ [23.51, 27.16]	24.52^a^ [22.97, 26.09]	22.94^b^ [21.91, 23.97]	*0.00*
CAL	2.83 [2.72, 2.94]	0.88^a^ [0.59, 1.18]	2.18^a^ [2.06, 2.30]	2.68^b^ [2.52, 2.84]	3.28^c^ [3.13, 3.44]	*0.00*
PPD	2.53 [2.45, 2.62]	1.89^a^ [1.80, 1.97]	2.17^a^ [2.07, 2.27]	2.41^b^ [2.28, 2.53]	2.86^c^ [2.73, 299]	*0.00*
% PPD ≥ 4 mm	9.30 [7.81, 10.79]	0.10^a^ [0.01, 0.26]	0.22^b,c^ [0.02, 0.40]	7.03^c^ [3.57, 9.49]	15.59^d^ [13.22, 17.95]	*0.00*
% PPD 5‐6 mm	7.71 [6.51, 8.90]	0^a^ [0, 0]	0.18^b,c^ [0.09, 0.39]	6.19^c^ [3.03,7.35]	12.83^d^ [10.93, 14.72]	*.00*
% PPD > 6 mm	4.03 [0.19, 8.24]	0^a^ [0, 0]	0^a,b^ [0, 0]	9.67^b^ [8.19, 27.29]	3.41^c^ [2.28, 4.55]	*0.00*
Furcation involvement, yes^‡^	76 (32.34%)	0^a^ (0%)	0^a^ (0%)	9^a^ (16.07%)	67^b^ (56.78%)	*0.00*
Mobility, yes	91 (38.72%)	0^a^ (0%)	1^a^ (3.70%)	17^a^ (30.36%)	73^b^ (61.86%)	*0.00*
Number of bleeding pockets^§^	7.32 [5.98, 8.65]	0^a^ [0, 0]	0^b,c^ [0, 0]	4.16^c^ [2.55, 5.76]	12.26^d^ [10.06, 14.46]	*0.00*
FMPS	50.56 [47.45, 53.68]	32.47^a^ [27.05, 37.89]	55.66^b^ [46.59, 64.74]	50.64^b^ [44.41, 56.87]	54.57^b^ [50.03, 59.11]	*0.02*
FMBS	28.45 [26.17, 30.74]	14.97^a^ [11.29, 18.64]	22.70^a,b^ [16.90, 28.50]	28.13^b,c^ [23.15, 33.09]	33.81^c^ [30.65, 36.97]	*0.00*
Teeth lost for periodontitis, yes	76 (32.34%)	0^a^ (0%)	0^a^ (0%)	0^a^ (0%)	76^b^ (64.41%)	*0.00*
Grade^¶^						
None	34 (14.45%)	34 (100%)	0 (0%)	0 (0%)	0 (0%)	*0.00*
A	21 (8.94%)	0^a^ (0%)	12^b^ (44.44%)	5^c^ (8.93%)	4^d^ (3.39%)	
B	111 (47.23%)	0^a^ (0%)	11^b^ (40.74%)	43^c^ (76.78%)	57^d^ (48.31%)	
C	69 (29.36%)	0^a^ (0%)	4^b^ (14.81%)	8^c^ (14.29%)	57^d^ (48.31%)	
Extent^¶^						
None	34 (14.45%)	34 (100%)	0 (0%)	0 (0%)	0 (0%)	0.96
Localized	104 (44.25%)	0 (0%)	15 (55.56%)	28 (50%)	61 (51.69%)	
Generalized	97 (41.30%)	0 (0%)	12 (44.44%)	28 (50%)	57 (48.31%)	
*Domiciliary plaque control*						
Brushing frequency						
Not performed	4 (1.70%)	1 (2.94%)	0 (0%)	0 (0%)	3 (2.54%)	0.06
Occasionally	46 (19.57%)	3 (8.82%)	3 (11.11%)	7 (12.50%)	33 (27.97%)	
Every day	185 (78.72%)	30 (88.24%)	24 (88.89%)	49 (87.50%)	82 (69.49%)	
Toothbrush type, powered	122 (51.91%)	15 (44.12%)	14 (51.85%)	30 (53.57%)	63 (53.39%)	0.47
Interdental cleaning (IC)					0.23	
Not performed	75 (31.91%)	15 (44.12%)	9 (33.33%)	18 (32.14%)	34 (28.81%)	
Interdental floss	51 (21.70%)	9 (26.47%)	7 (25.93%)	12 (21.43%)	23 (19.49%)	
Interproximal brushes	109 (45.99%)	10 (29.41%)	11 (40.74%)	26 (46.43%)	61 (51.69%)	
Frequency of IC						0.72
Not performed	75 (31.91%)	14 (41.18%)	9 (33.33%)	17 (30.36%)	35 (29.66%)	
Occasionally	48 (20.43%)	6 (17.65%)	4 (14.81%)	9 (16.07%)	29 (24.58%)	
Every day	112 (47.66%)	14 (41.18%)	14 (51.85%)	30 (53.57%)	54 (45.76%)	

*Note*: results of continuous variables are reported as mean [95% confidence interval]; results of binary and categorical variables are expressed as number of observations (proportion).

Abbreviations: aMed, alternate Mediterranean diet score; BMI, body mass index; CAL, clinical attachment level; FMBS, full‐mouth bleeding score; FMPS, full‐mouth plaque score; IC, interdental cleaning.; PA, physical activity; PPD, probing depth.

Values with different superscript letters are different at the 5% level.

*P‐value of the Kruskal Wallis or Fisher's exact test for patients’ characteristics across the four subgroups.

† High aMed if aMed >4; Low aMed if aMed <5.

‡ Class II/III furcation involvement according to the classification of Hamp et al.

§ Defined as the number of sites with probing depth ≥ 5 mm and positive to bleeding on probing.

¶ According to the 2018 EFP/AAP classification.

### 3.2 Outcome data

#### 3.2.1 Adherence to MD and periodontitis

High adherence to MD was significantly associated to a lower prevalence of Stage III/IV periodontitis (29.66%) compared to those with low adherence (70.34%) (Table 2). Other biometric periodontal variables (i.e., %PPD >4 mm, %PPD 5 to 6 mm, furcation involvement, tooth mobility, number of bleeding pockets, teeth lost for periodontal causes) were reported to be significantly worse in individuals with low MD adherence (Table 2). High aMed scores as well as the frequent consumption of specific MD foods resulted to be associated with lower odds of periodontitis occurrence as well as Stage III/IV periodontitis, even after adjusting for age, sex, smoking and brushing frequency (Supplementary Table S3 in online *Journal of Periodontology*; Table 3).

**TABLE 2:** Patients’ characteristics by adherence to Mediterranean diet (aMed) and physical activity level

	aMed			PA level		
	Low (0‐4)	High (5‐9)	*P*‐value*	Low/moderate	High	*P‐value* *
Variable	n = 117	n = 118		n = 168	n = 67	
Age	53.63 [50.88, 56.37]	54.17 [51.54, 56.80]	0.93	54.52 [52.36, 56.68]	52.39 [48.45, 56.23]	0.35
BMI	25.90 [25.02, 26.78]	25.09 [24.22, 25.96]	0.11	25.96 [25.19, 26.72]	24.32 [23.33, 25.31]	*0.01*
Sex, females	56 (47.86%)	80 (67.80%)	*0.00*	105 (62.50%)	31 (46.27%)	*0.03*
Occupation						
Unemployed	26 (22.41%)	16 (13.56%)	0.15	31 (18.56%)	11 (16.42%)	0.93
Employed	65 (56.03%)	68 (57.63%)		94 (56.29%)	39 (58.21%)	
Retired	25 (21.55%)	34 (28.81%)		42 (25.15%)	17 (25.37%)	
Education						
Elementary/middle school	43 (37.07%)	26 (22.03%)	*0.01*	52 (30.95%)	17 (25.76%)	0.71
High school	53 (45.69%)	54 (45.76%)		76 (45.24%)	31 (46.97%)	
College or more	20 (17.24%)	38 (32.20%)		40 (23.81%)	18 (27.27%)	
Smoking						
Never	47 (40.17%)	60 (50.85%)	0.25	70 (41.67%)	37 (55.22%)	0.18
Former	36 (30.77%)	31 (26.27%)		51 (30.36%)	16 (23.88%)	
Smoker	34 (29.06%)	27 (22.88%)		47 (27.98%)	14 (20.90%)	
Familiarity for periodontitis, yes	42 (35.90%)	45 (38.14%)	0.79	65 (38.69%)	22 (32.84%)	0.46
*Oral health status*						
Grade^c^						
None	34 (14.45%)	0.67	34 (14.45%)	0.86		
A	3 (1.28%)	18 (7.66%)		10 (4.26%)	11 (4.68%)	
B	55 (23.41%)	56 (23.83%)		78 (33.19%)	33 (14.05%)	
C	43 (18.30%)	26 (11.07%)		54 (22.98%)	15 (6.39%)	
Extent^c^						
None	34 (14.45%)	0.67	34 (14.45%)	0.32		
Localized	52 (22.13%)	52 (22.13%)		78 (33.19%)	26 (11.07%)	
Generalized	48 (20.43%)	49 (20.86%)		66 (28.09%)	31 (13.20%)	
Number of teeth	23.85 [22.81, 24.89]	24.48 [23.55, 25.41]	0.48	24.05 [23.24, 24.88]	24.52 [23.09, 25.79]	0.48
CAL	2.96 [2.79, 3.14]	2.70 [2.57, 2.83]	0.06	2.88 [2.75, 3.02]	2.69 [2.52, 2.86]	
PPD	2.62 [2.49, 2.75]	2.44 [2.34, 2.55]	0.06	2.56 [2.46, 2.67]	2.45 [2.32, 2.59]	0.32
% PPD >4 mm	11 [8.74, 13.21]	7.61 [5.69, 9.54]	*0.02*	10.21 [8.32, 12.10]	7.01 [4.87, 9.15]	0.12
% PPD 5‐6 mm	9.12 [7.30, 10.95]	6.31 [4.76, 7.84]	*0.02*	8.58 [7.04, 10.13]	5.51 [3.94, 7.06]	0.09
% PPD >6 mm	5.57 [2.81, 13.96]	2.46 [1.37, 3.55]	0.18	5 [0.01, 10.90]	1.56 [0.5, 2.64]	0.39
Furcation involvement, yes^a^	51 (43.59%)	25 (21.19%)	*0.00*	60 (35.71%)	16 (23.88%)	0.09
Mobility, yes	55 (47.01%)	36 (30.51%)	*0.01*	77 (45.83%)	14 (20.90%)	*0.00*
Number of bleeding pockets^b^	9.18 [6.95, 11.41]	5.46 [4.03, 6.89]	0.03	7.91 [6.25, 9.58]	5.83 [3.69, 7.97]	0.16
FMPS	51.92 [47.45, 56.39]	49.22 [44.82, 53.61]	0.48	51.19 [47.27, 55.11]	48.98 [44.08, 53.88]	0.61
FMBS	29.78 [26.57, 33.01]	27.14 [23.86, 30.41]	0.18	28.69 [25.88, 31.51]	27.85 [23.93, 31.76]	0.96
Teeth lost for periodontitis, yes	47 (40.17%)	29 (24.58%)	*0.01*	59 (35.12%)	17 (25.37%)	0.16
*Domiciliary plaque control*						
Brushing frequency						
Not performed	3 (2.56%)	1 (0.85%)	0.06	4 (2.38%)	0 (0%)	0.39
Occasionally	29 (24.79%)	17 (14.41%)		35 (20.83%)	11 (16.42%)	
Every day	85 (72.65%)	100 (84.75%)		129 (76.79%)	56 (83.58%)	
Toothbrush type, powered	62 (52.99%)	60 (50.85%)	0.79	90 (53.57%)	32 (47.76%)	0.47
Interdental cleaning (IC)						
Not performed	43 (36.75%)	32 (27.12%)	0.06	58 (34.52%)	17 (25.37%)	0.51
Interdental floss	29 (24.79%)	22 (18.64%)		36 (21.43%)	15 (22.39%)	
Interproximal brushes	45 (38.46%)	64 (54.24%)		74 (44.05%)	35 (42.24%)	
Frequency of IC						
Not performed	44 (37.61%)	31 (26.27%)	0.06	58 (34.52%)	17 (25.37%)	0.37
Occasionally	28 (23.93%)	20 (16.95%)		32 (19.05%)	16 (23.88%)	
Every day	45 (38.46%)	67 (56.78%)		78 (46.43%)	34 (50.75%)	

*Note*: results of continuous variables are reported as mean [95% confidence interval]; results of binary and categorical variables are expressed as number (proportion).

Abbreviations: aMed, alternate Mediterranean diet score; BMI, body mass index; CAL, clinical attachment level; FMBS, full‐mouth bleeding score; FMPS, full‐mouth plaque score; IC, interdental cleaning.; PA, physical activity; PPD, probing depth.

*P‐value of the Mann Whitney or Fisher's exact test for patients’ characteristics across the subgroups of diet and physical activity; *P *< 0.05.

^a^ Class II/III furcation involvement according to the classification of Hamp et al.

^b^ Defined as the number of sites with probing depth ≥ 5 mm and positive to bleeding on probing.

^c^ According to the 2018 EFP/AAP classification.

**TABLE 3:** Association between aMed and physical activity level with Stage III/IV periodontitis

	ORs for Stage III/IV periodontitis							
		95% CI				95% CI		
Variable	Crude ORs	Lower	Upper	*P*‐value*	Adjusted^a^ ORs	Lower	Upper	*P*‐value*
Low/moderate PA level	1.83	1.04	3.26	*0.04*	1.65	0.84	3.28	0.42
Low aMed^b^	8.47	4.20	17.09	*0.00*	5.63	3.21	9.84	*0.00*
*aMed and PA level*								
Low aMed, low/moderate PA	7.69	3.03	19.47	*0.01*	6.40	2.94	13.91	*0.01*
Low aMed, high PA	7.66	1.98	29.95	*0.00*	3.30	1.15	9.50	*0.03*
High aMed^b^, low/moderate PA	0.96	0.44	2.20	0.97	0.85	0.32	2.23	0.74
High aMed, high PA	REF.							
*aMed components*								
Wholegrain products	0.33	0.19	0.56	*0.00*	0.25	0.13	0.47	*0.00*
Vegetables	0.47	0.28	0.80	*0.00*	0.43	0.23	0.80	*0.01*
Fruits	0.56	0.33	0.94	*0.03*	0.37	0.19	0.79	*0.00*
Olive oil	0.40	0.19	0.82	*0.01*	0.30	0.15	0.82	*0.00*
Wine	0.67	0.39	1.16	0.15	0.62	0.32	1.19	0.15
Red meat and meat products	0.67	0.37	1.15	0.07	0.69	0.37	1.27	0.24
Fish	0.36	0.21	0.63	0.*00*	0.38	0.20	0.71	*0.00*
Dried fruits	0.33	0.19	0.58	0.*00*	0.35	0.15	0.59	*0.02*
Pulses	0.54	0.31	0.96	0.*04*	0.39	0.22	0.73	*0.00*

Abbreviations: aMed, alternate Mediterranean diet score; CI, confidence interval; ORs, odds ratios; PA, physical activity; REF., reference category.

*P < 0.05.

^a^ Adjusted for age, sex, smoking, and brushing frequency.

^b^ High aMed if aMed >4; Low aMed if aMed <5.

#### 3.2.2 Physical activity and periodontitis

Around 1/3 of participants reached a high PA level, while the other 2/3 were in the low/moderate PA level category (Table 2). A high PA level was significantly associated with a lower prevalence of Stage III/IV periodontitis (22.03%) when compared to low/moderate PA (77.97%). A low/moderate PA level was not associated with periodontitis occurrence (Supplementary Table S3). Conversely, a low/moderate PA level increased the odds of having Stage III/IV periodontitis, but not after adjustments (OR = 1.65 [0.84, 3.28], *P *= 0.42) (Table 3).

#### 3.2.3 Combining adherence to MD and physical activity

Around 40% of participants belonged to the “low aMed, low/moderate PA” subgroup, while fewer subjects belonged to the other three categories (Table 4). The prevalence of Stage III/IV periodontitis was significantly higher in the subgroups “low aMed, low/moderate PA” (73.68%) and “low aMed, high PA” (59.09%), when compared to the “high aMed, low/moderate PA” (30.14%) and the “high aMed, high PA” (28.89%); the periodontal condition was found to be significantly worse in the two former compared to the two latter categories (*P *= 0.04). The proportion of subjects who reported having lost teeth for periodontitis almost doubled when shifting from the “high aMed, high PA” (24.44%) to the “low aMed, low/moderate PA” (43.16%) subgroup (*P *= 0.04). Subgroups “low aMed, low/moderate PA” and “low aMed, high PA” resulted in significantly positive adjusted (Table 3) ORs for Stage III/IV periodontitis, while only the former was positively associated with periodontitis occurrence (Supplementary Table S3) (OR = 1.53 [1.04, 1.74], *P *= 0.01). Moreover, results of the final model are shown in Table 5. The presence of low MD adherence, irrespective of PA level, increased the odds of Stage III/IV periodontitis by 9 (low/moderate PA level) and 10 (high PA level) times in both cases (*P *= 0.00). Age (OR = 1.08 [1.05, 1.11], *P *= 0.00), the presence of smoking habit (OR = 2.16 [1.02, 4.91], *P *= 0.04) and familiarity for periodontitis (OR = 2.13 [1.08, 4.20], *P *= 0.029) were significant predictors in this observation. The model resulted statistically significant (*P *= 0.00) with a pseudo R^2^ of around 30%. Moreover, results from the mediation analysis demonstrated that about 9% of the effect of the combination between MD and PA on Stage III‐IV periodontitis was mediated by BMI; this mediation was shown to be partial (Sobel test, *P*‐value = 0.064) (see Supplementary Table S4 and Supplementary Figure S1 in online *Journal of Periodontology*).

**TABLE 4:** Patients’ characteristics summarized by the combination of adherence to Mediterranean diet (aMed score) and physical activity level

	Low aMed, low/moderate PA	Low aMed, high PA	High aMed, low/moderate PA	High aMed, high PA	*P*‐value*
Variable	n = 95	n = 25	n = 69	n = 46	
*Sociodemographic characteristics*					
Age	55.26 [52.36, 58.15]	46.58 [39.22, 53.94]	53.56 [50.26, 56.86]	55.15 [50.64, 59.66]	0.26
BMI	26.06^,d^ [25.10, 27.02]	25.22^a,c,d^ [22.95, 27.49]	25.81^b,c^ [24.57, 27.07]	23.88^a^ [22.87, 24.90]	*0.04*
Sex, females	50^c^ (52.63%)	6^b^ (27.27%)	55^a^ (75.34%)	25^a,b,c^ (55.56%)	*0.00*
Occupation					
Unemployed	22 (23.40%)	4 (18.18%)	9 (12.33%)	7 (15.56%)	0.48
Employed	50 (53.19%)	15 (68.18%)	44 (60.27%)	24 (53.33%)	
Retired	22 (23.40%)	3 (13.64%)	20 (27.40%)	14 (31.11%)	
Education					
Elementary/middle school	35 (36.84%)	8 (38.10%)	17 (23.29%)	9 (20%)	0.66
High school	42 (44.21%)	11 (52.38%)	34 (46.58%)	20 (44.44%)	
College or more	18 (18.95%)	2 (9.52%)	22 (30.14%)	16 (35.56%)	
Smoking					
Never	37 (38.95%)	10 (45.45%)	33 (45.21%)	27 (60%)	0.69
Former	31 (32.63%)	5 (22.73%)	20 (27.40%)	11 (24.44%)	
Smoker	27 (28.42%)	7 (31.82%)	20 (27.40%)	7 (15.56%)	
Familiarity for periodontitis, yes	36 (37.89%)	6 (27.27%)	29 (39.73%)	16 (35.56%)	0.46
*Oral health status*					
Periodontitis^†^					
Healthy‐gingivitis	11 (32.35%)^a^	6 (17.65%)^a^	13 (38.24%)^b^	4 (11.76%)^b^	*0.04*
Stage I	1 (3.70%)^a^	0 (0%)^a^	14 (51.85%)^b^	12 (44.44%)^b^	
Stage II	13 (23.21%)^a^	3 (5.36%)^a^	24 (42.86%)^b^	16 (28.57%)^b^	
Stage III/IV	70 (73.68%)^a^	13 (59.09%)^a^	22 (30.14%)^b^	13 (28.89%)^b^	
Grade^†^					
None	14 (14.74%)	6 (24%)	12 (17.39%)	2 (4.35%)	0.32
A	3 (3.16%)	0 (0%)	7 (10.15%)	11 (23.91%)	
B	42 (44.21%)	13 (52%)	33 (47.82%)	23 (50%)	
C	36 (37.89%)	6 (24%)	17 (24.64%)	10 (21.74%)	
Extent^†^					
None	34 (14.45%)	0.12			
Localized	43 (18.30%)	9 (3.84%)	35 (14.89%)	17 (7.23%)	
Generalized	41 (17.45%)	7 (2.98%)	25 (10.65%)	24 (10.21%)	
Number of teeth	23.74 [22.60, 24.89]	24.31 [21.58, 27.04]	24.46 [23.28, 25.64]	24.51 [22.92, 26.09]	0.76
CAL (mm)	3.02 [2.82, 3.22]	2.71 [2.31, 3.11]	2.70 [2.52, 2.89]	2.68 [2.50, 2.86]	0.07
PPD (mm)	2.64 [2.50, 2.79]	2.52 [2.20, 2.84]	2.46 [2.31, 2.61]	2.42 [2.29, 2.56]	0.18
% PPD > 4 mm	11.64 [9.07, 14.20]	8.22 [3.37, 13.07]	8.35 [5.54, 11.11]	6.42 [4.15, 8.68]	0.07
% PPD 5‐6 mm	9.92 [7,79, 12.05]^a^	5.68 [2.65, 8.71]^b^	6.84 [4.61, 9.07]^b^	5.42 [3.54, 7.30]^b^	*0.04*
% PPD > 6 mm	2.42 [1.26, 3.57]	2.63 [0.5, 5.81]	8.36 [5.26, 22]	1.04 [0.45, 1.63]	0.43
Furcation involvement, yes^‡^	43 (45.26%)^a^	8 (36.36%)^a,c,d^	17 (23.29%)^b,c^	8 (17.78%)^b,d^	*0.00*
Mobility, yes	50 (52.63%)^a^	5 (22.73%)^b,c^	27 (36.99%)^a,c^	9 (20%)^b,c^	*0.00*
Number of bleeding pockets^§^	9.68 [7.16, 12.20]	7.05 [2.02, 12.07]	5.60 [3.68, 7.52]	5.24 [3.05, 7.43]	0.07
FMPS	53.15 [48.12, 58.19]	46.59 [36.43, 56.74]	48.64 [42.34, 54.94]	50.15 [44.52, 55.78]	0.59
FMBS	30.48 [26.80, 34.16]	26.77 [20.00, 33.54]	26.39 [21.97, 30.76]	28.37 [23.40, 33.35]	0.35
Teeth lost for periodontitis, yes	41 (43.16%)^a^	6 (27.27%)^a,c^	18 (24.66%)^b,c^	11 (24.44%)^c^	*0.04*
*Domiciliary plaque control*					
Brushing frequency					
Not performed	3 (3.16%)	0 (0%)	1 (1.37%)	0 (0%)	0.31
Occasionally	25 (26.32%)	4 (18.18%)	10 (13.70%)	7 (15.56%)	
Every day	67 (70.53%)	18 (81.82%)	62 (84.93%)	38 (84.44%)	
Toothbrush type, powered	53 (55.79%)	9 (40.91%)	37 (50.68%)	23 (51.11%)	0.63
Interdental cleaning (IC)					
Not performed	36 (37.89%)	7 (31.82%)	22 (30.14%)	10 (22.22%)	0.16
Interdental floss	21 (22.11%)	8 (36.36%)	15 (20.55%)	7 (15.56%)	
Interproximal brushes	38 (40%)	7 (31.82%)	36 (49.32%)	28 (62.22%)	
Frequency of IC					
Not performed	37 (38.95%)	7 (31.82%)	21 (28.77%)	10 (22.22%)	0.09
Occasionally	20 (21.05%)	8 (36.36%)	12 (16.44%)	8 (17.78%)	
Every day	38 (40%)	7 (31.82%)	40 (54.79%)	27 (60%)	

*Note*: results of continuous variables are reported as mean [95% confidence interval]; results of binary and categorical variables are expressed as number (proportion).

Abbreviations: aMed, alternate Mediterranean diet score; CAL, clinical attachment level; FMBS, full‐mouth bleeding score; FMPS, full‐mouth plaque score; IC, interdental cleaning; PA, physical activity; PPD, probing depth.

Values with different superscript letters are different at the 5% level.

*P‐value of the Kruskal Wallis or Fisher's exact test for patients’ characteristics across the four subgroups; *P *< 0.05.

† According to the 2018 EFP/AAP classification.

‡ Class II/III furcation involvement according to the classification of Hamp et al.

§ Defined as the number of sites with probing depth ≥ 5 mm and positive to bleeding on probing.

**TABLE 5:** Multivariate logistic regression analysis for the prediction of Stage III/IV periodontitis by measures of the combination of adherence to Mediterranean diet (aMed score) and physical activity, and sociodemographic characteristics

Best model (AUC = 0.853, AIC = 212.4, BIC = 252.5)						
LR chi^2^	Prob >chi^2^	Pseudo R^2^				
97.61	0.00	0.2996			95% CI	
Stage III/IV periodontitis	OR	SE	Z	*P*‐value*	Lower	Upper
High aMed^a^, high PA	REF.					
High aMed, low/moderate PA	1.06	0.49	0.12	0.90	0.42	2.65
Low aMed^a^, high PA	9.26	6.28	3.28	*0.00*	2.45	35.03
Low aMed, low/moderate PA	10.23	4.88	4.86	*0.00*	4.01	26.09
Age	1.08	0.01	5.79	*0.00*	1.05	1.11
*Smoking*						
Never	REF.					
Former	1.23	0.49	0.57	0.56	0.58	2.69
Smoker	2.16	0.90	1.83	*0.04*	1.02	4.91
Familiarity for periodontitis	2.13	0.74	2.18	*0.029*	1.08	4.20
Comorbidities^b^	1.59	0.72	1.00	0.30	0.64	3.81
*_cons*	0.00	0.00	‐6.43	0.00	0.00	0.02

Abbreviations: AIC, Akaike information criterion; aMed, alternate Mediterranean diet score; AUC, area under the curve; BIC, Bayesian information criterion; CI, confidence interval; LR, likelihood ratio; PA, physical activity; REF., reference category.

*P < 0.05.

^a^ High aMed if aMed >4; Low aMed if aMed <5.

^b^ Presence of at least one comorbidity (i.e., diabetes, rheumatoid arthritis, inflammatory bowel diseases, osteoporosis).

## 4 DISCUSSION

### 4.1 Summary of findings

In the current study, the odds of Stage III/IV periodontitis were found to be double in subjects with low/moderate PA level compared to those with high PA level and almost 6 times higher in subjects with low MD adherence compared to those with high adherence. The final regression model demonstrated how the combination of poor MD adherence and PA level led to 10‐time increased odds of Stage III/IV periodontitis. Age, smoking and familiarity for periodontitis resulted as additional predictors for Stage III/IV periodontitis. Moreover, the significant mediating effect of BMI accounted for around 9% of the direct effect of the combination between MD and PA on the occurrence of Stage III/IV periodontitis.

### 4.2 Dietary and physical activity assessments

This is the first study formulating the hypothesis of a significant association between PA level and MD adherence with periodontitis severity. Overall, high values of physical inactivity were detected in the present cohort (71%), maybe due to the presence of comorbidities in around 20% of the included individuals. High PA levels were associated with lower prevalence of Stage III/IV periodontitis, consistently to recent metadata showing how physically active patients are 22% less likely to have periodontitis when compared to those physically inactive.^14^

A linear relationship between decreasing values of aMed scores and the worsening of periodontal indices was also found, with around 70% of those with low MD adherence being affected by Stage III/IV periodontitis. Results from the present study are partially discordant from those of a previous report^9^ in which no differences in the biometric periodontal indices were found between subjects with high MD adherence compared to those with low adherence. This result may be due to the different target population investigated in both studies: while the former enrolled University students (mean age 20 years), participants from the current report were selected among those coming to a periodontal Unit of a public University Hospital in Italy (mean age 53 years). Consequently, the reported values of periodontitis prevalence widely differ (6.6% versus 85%).

Indeed, subjects with low MD adherence and low/moderate PA presented not only worse biometric and inflammatory parameters, but also a more frequent experience of tooth mobility and tooth loss for periodontitis, irrespective of domiciliary oral hygiene measures and plaque accumulation. Therefore, a reciprocal association between such environmental factors (MD and PA) and the periodontium can be speculated. Firstly, the clinical manifestations of severe periodontitis, such as advanced CAL, tooth loss and tooth mobility, hamper masticatory function up to a point that they can lead to dietary changes, characterized by a decrease in fibers, fruits and vegetable intake.^34^ In turn from a biological standpoint, the consumption of a Western diet, rich in white flour and processed meats, as opposed to MD, induces a state of low‐grade inflammation, contributing to the development of many NCDs, including periodontitis.^35^ Conversely, high MD adherence was demonstrated to be inversely related with Stage III/IV periodontitis, supposedly due to the synergistic anti‐inflammatory potential of the single MD components: wholegrain products demonstrated the strongest protective effect in the current study. In fact, a diet rich in wholegrains was associated with lower systemic inflammatory markers, such as C‐reactive protein, and decreased insulin resistance.^36^, ^37^ The improved insulin sensitivity could positively influence periodontal health by lowering the production of glycation end‐products, reducing oxidative stress and, therefore, by decreasing cytokines release. From a microbiological standpoint, an increased MD adherence was found to lead to a significant decrease in the salivary concentration of microorganisms such as *Porphyromonas gingivalis*, *Prevotella intermedia*, and *Treponema denticola* ^38^; therefore, the increased consumption of MD components may induce a microbial shift in the saliva, hence contributing to the host defense immunomodulation.^39^

On the other hand, low MD adherence and, in particular, a low consumption of wholegrain products, increases the odds of Stage III/IV periodontitis by almost 8 times plausibly by decreasing insulin sensitivity and increasing low‐grade systemic inflammation.^40^

The current study also hypothesized a positive synergistic effect of regular exercise, in conjunction with high MD adherence, on periodontal health. Even though the effects of MD are prominent when compared independently to those of PA, regular physical exercise was previously demonstrated to decrease the concentration of specific proinflammatory markers involved in the clinical manifestations of periodontitis.^41^

### 4.3 Final multivariate regression model

The final regression model highlights the ability of the combination between low MD adherence and low PA to increase the odds of Stage III/IV periodontitis by 10 times; nonetheless, every time MD adherence is below the selected cut‐off, the OR increases irrespective of PA level. Patient's lifestyle, in terms of diet and PA, seems to retain higher odds than smoking for Stage III/IV periodontitis. It can be speculated that such result may be due to the significant mediating effect exerted by BMI: indeed, low MD adherence and low PA level were previously correlated with higher BMI values,^42^ which in turn can potentially increase the odds for Stage III/IV periodontitis.^43^ Such results may be relevant to better understand the etiopathogenesis of periodontitis, but may also have critical implications from a therapeutical standpoint: Step 1 phase^44^ of periodontal therapy could be implemented by applying a holistic approach based on common risk factors^45^ not only for oral health, but also for other NCDs. Therefore, risk‐factor modification should encompass smoking cessation counseling and oral hygiene instructions on one side, but also the promotion of both regular exercise and MD adherence on the other. Thus, this holistic approach based on common risk‐factor modification could significantly ameliorate both the treatment efficacy and long‐term management of lifestyle‐related pathologies, and also result in reduced healthcare costs.

### 4.4 Strengths and limitations

This is the first study investigating the association between the combination of MD adherence and PA level and Stage III/IV periodontitis. Diet and PA assessments were carried out using reliable and validated tools for the selected sample. In fact, the 15‐item questionnaire used to evaluate MD adherence (QueMD) was the first one to be developed for the Italian population and validated against a Food Frequency Questionnaire.^23^ Moreover, PA levels were drawn from the results of the Short version of the IPAQ, whose reproducibility and validity for the Italian population were demonstrated.^26^ Altogether, these factors significantly contribute to the internal validity of the study.

The current study presents some limitations. First of all, the cross‐sectional design does not allow for a longitudinal evaluation regarding the cause‐effect relationship between the exposure and the outcome, and therefore it can be used only to build a hypothesis. Indeed, this design does not allow also to properly investigate reverse causality (i.e., effect of periodontitis on nutrition capability and dietary changes); moreover, no molecular parameters supporting the biological plausibility of such association were registered. Secondly, due to the lack of studies regarding the combined effect of MD and PA on periodontal health, the sample size calculation was instead performed based on the prevalence of periodontitis; therefore, the potential lack of power could explain the absence of association for PA in the multivariate models. Third, the assessments of the exposure (i.e., administration of questionnaires) and the outcome (periodontal chart) were performed by the same examiners, who therefore were not blinded during outcome assessment. Furthermore, all participants lived in the urban or suburban areas near Siena (Italy), where the vast majority of adults are Caucasian; hence, any variability in the outcome related to ethnicity may not have been detected. Moreover, given that the study population was selected among patients coming to a public Unit of Periodontology, the risk of selection bias could not be ruled out. Overall, these factors may reduce the generalizability of the study.

## 5 CONCLUSION

The present study demonstrated that individuals conducting a lifestyle characterized by low adherence to MD are more than 9 times more likely to have severe manifestations of periodontitis, irrespective of PA level. Further research is needed to elucidate the periodontal response to the implementation of Step 1 phase of periodontal treatment with nutritional and PA counseling sessions.

## Supporting information


**Supplementary Appendix**: Assessment methods of sociodemographic characteristics.

**Supplementary Figure 1**: Mediating effect of BMI on the impact of combined aMed/PA on Stage III/IV periodontitis.

**Supplementary Table 1**: Questionnaires regarding adherence to Mediterranean Diet (QueMD) and physical activity (International Physical Activity Questionnaire).

**Supplementary Table 2**: *Post‐hoc* power analysis for the association between Stage III/IV periodontitis and each evaluated risk indicator.

**Supplementary Table 3**: Association between aMed and physical activity level with periodontitis case.

**Supplementary Table 4**: Results of the mediation analysis of BMI on the effect of the combination of diet and physical activity on the presence of Stage III/IV periodontitis.

## Data Availability

The data that support the findings of this study are available upon reasonable request from the corresponding author. The data are not publicly available due to privacy or ethical restrictions.

## Footnotes

* UNC 15 probe, Hu‐Friedy Group, Chicago, Illinois.

† STATA BE, version 17, StataCorp LP, College Station, Texas.
