# Supplementary material for: Adherence to Mediterranean diet, physical activity level, and severity of periodontitis: Results from a university‐based cross‐sectional study
Source: J Periodontol. 2022 Feb 25;93(8):1218–32. doi: 10.1002/JPER.21-0643 (PMC9544461; doi:10.1002/JPER.21-0643)
Supplement: Supplementary file 1 — Supplementary Appendix: Assessment methods of sociodemographic characteristics. [file JPER-93-1218-s003.docx]

**Supplementary Appendix.** Assessment methods of socio-demographic characteristics.

As socio-demographic characteristics, the following variables were considered: age, Body Mass Index (BMI), gender, occupation, education, smoking, familiarity for periodontitis, presence of comorbidities (diabetes, rheumatoid arthritis, inflammatory bowel diseases and osteoporosis), oral hygiene habits.

- The BMI was calculated as weight (kilograms)/height (meters^2^). Weight was measured with a scale in kilograms, and height was measured with a stadiometer in meters.

- Occupation was categorized as unemployed, employed or retired.

- Educational level was categorized as the highest diploma obtained (primary/middle school, high school, college or more).

- Smoking was self-reported and categorized as smoker, former smoker or never smoked.

- Familiarity for periodontitis was self-reported and categorized as yes/no.

- As for the presence of comorbidities (diabetes, rheumatoid arthritis, inflammatory bowel diseases and osteoporosis), the variable was categorized as: affected, not affected or having familiarity for the disease. The presence of the specific disease was self-reported but confirmed by checking the patient’s medical report. Familiarity for the specific disease was self-reported.

- Information regarding oral hygiene habits was self-reported. Brushing frequency was categorized as not performed, performed occasionally, every day. Toothbrush type was categorized as powered or manual. The device used for interdental cleaning was categorized as using interproximal brushes, interdental floss or not performed. The frequency of interdental cleaning was categorized as not performed, occasionally or performed every day.
