# Supplementary figures and images for: Adherence to Mediterranean diet, physical activity level, and severity of periodontitis: Results from a university‐based cross‐sectional study
Source: J Periodontol. 2022 Feb 25;93(8):1218–32. doi: 10.1002/JPER.21-0643 (PMC9544461; doi:10.1002/JPER.21-0643)

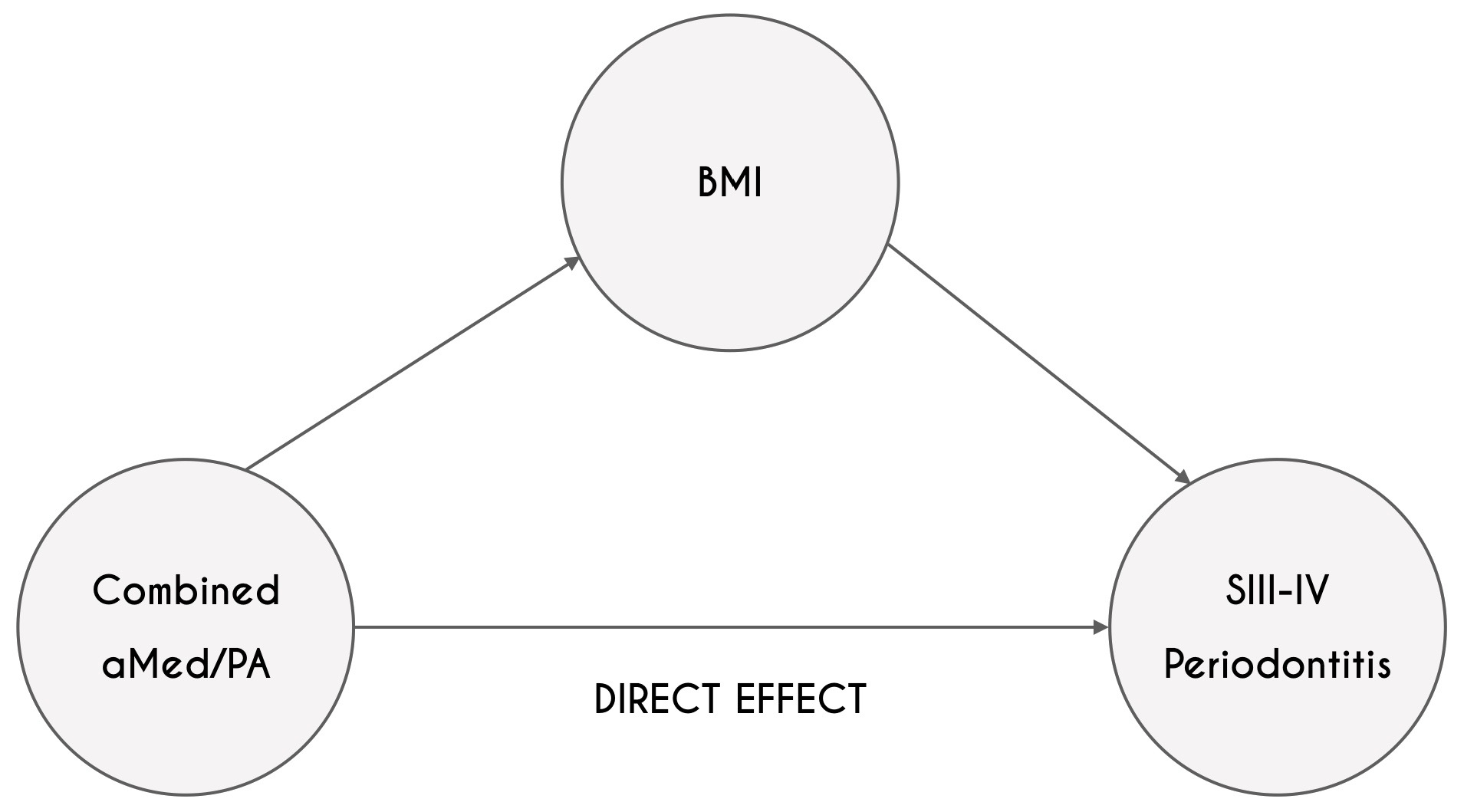

Supplement: Supplementary file 2 — Supplementary Figure 1: Mediating effect of BMI on the impact of combined aMed/PA on Stage III/IV periodontitis. [file JPER-93-1218-s002.jpg]
