# Supplementary material for: Adherence to Mediterranean diet, physical activity level, and severity of periodontitis: Results from a university‐based cross‐sectional study
Source: J Periodontol. 2022 Feb 25;93(8):1218–32. doi: 10.1002/JPER.21-0643 (PMC9544461; doi:10.1002/JPER.21-0643)
Supplement: Supplementary file 3 — Supplementary Table 1: Questionnaires regarding adherence to Mediterranean Diet (QueMD) and physical activity (International Physical Activity Questionnaire). [file JPER-93-1218-s001.docx]

**Supplementary Table 1**: questionnaires regarding adherence to Mediterranean Diet (QueMD) and physical activity (International Physical Activity Questionnaire).

| **Adherence to Mediterranean diet (QueMD)** | | | | | | | | | | | | | | | | | | | | | | |
| --- | --- | --- | --- | --- | --- | --- | --- | --- | --- | --- | --- | --- | --- | --- | --- | --- | --- | --- | --- | --- | --- | --- |
| ***How often do you normally consume a portion of the following foods?*** | | | | | | | | | | | | | | | | | | | | | | |
| **Food Items** | **Daily frequency of consumption** | | | | | | | | | | | | | | | | | | | | | |
|  | **Reference**  **portions** | | **Never**  **or seldom** | | **<1 per**  **day** | | | | | | **1 per**  **day** | | | | | | **2 per**  **day** | | | | | **≥3 per**  **day** |
| 1. Wholegrain pasta or rice | 80 g | 🞎 | | 🞎 | | | | 🞎 | | | | | | | | 🞎 | | | | | | 🞎 |
| 2. Vegetables, all type (raw and cooked) | 200 g  80 g (salad) | 🞎 | | 🞎 | | | | 🞎 | | | | | | | | 🞎 | | | | | | 🞎 |
| 3. Fruits, all types fresh and fresh juices | 150 g | 🞎 | | 🞎 | | | | 🞎 | | | | | | | | 🞎 | | | | | | 🞎 |
| 4. Milk and yoghurt | 125 g | 🞎 | | 🞎 | | | | 🞎 | | | | | | | | 🞎 | | | | | | 🞎 |
|  |  | **Never or seldom** | | **<1 per day** | | | | **1-2 per day** | | | | | | | | **3-4 per day** | | | | | | **≥5 per day** |
| 5. Wholegrain bread and substitutes | 50 g (1-2 slices) | 🞎 | | 🞎 | | | | 🞎 | | | | | | | | 🞎 | | | | | | 🞎 |
| 6. Olive oil to cook and to dress | 10 ml (1 spoon) | 🞎 | | 🞎 | | | | 🞎 | | | | | | | | 🞎 | | | | | | 🞎 |
| 7. Butter, margarine or cooking cream | 10 g (1 spoon) | 🞎 | | 🞎 | | | | 🞎 | | | | | | | | 🞎 | | | | | | 🞎 |
| 8. Wine (white and red) | 125 ml (1 glass) | 🞎 | | 🞎 | | | | 🞎 | | | | | | | | 🞎 | | | | | | 🞎 |
|  |  | **Weekly frequency of consumption** | | | | | | | | | | | | | | | | | | | | |
|  |  | **Never or seldom** | | **<1 per**  **week** | | | | **1-3 per**  **week** | | | | | | | | **4-6 per**  **week** | | | | | | **≥7 per**  **week** |
| 9. Red meat (beef, veal, pork), meat products | 100 g (raw meat)  50 g (meat products) | 🞎 | | 🞎 | | | | 🞎 | | | | | | | | 🞎 | | | | | | 🞎 |
| 10. White meat (chicken, turkey, rabbit) | 100 g | 🞎 | | 🞎 | | | | 🞎 | | | | | | | | 🞎 | | | | | | 🞎 |
| 11. Carbonated and/or sugar-sweetened beverages | 200 ml (1 glass) | 🞎 | | 🞎 | | | | 🞎 | | | | | | | | 🞎 | | | | | | 🞎 |
| 12. Manufactured sweets, pastries, biscuits, creams… | 100 g | 🞎 | | 🞎 | | | | 🞎 | | | | | | | | 🞎 | | | | | | 🞎 |
|  |  | **Never or seldom** | | **<1 per**  **week** | | | | **1 per**  **week** | | | | | | | | **2-3 per**  **week** | | | | | | **≥4 per**  **week** |
| 13. Fish (fresh or frozen) or sea foods | 150 g (fish)  50 g (fish products) | 🞎 | | 🞎 | | | | 🞎 | | | | | | | | 🞎 | | | | | | 🞎 |
| 14. Dried fruits (nuts, almonds, hazelnuts) | 30 g (1 fist) | 🞎 | | 🞎 | | | | 🞎 | | | | | | | | 🞎 | | | | | | 🞎 |
| 15. Pulses (chickpeas, lentils, peas, beans) | 50 g (dried)  150 g (canned/raw) | 🞎 | | 🞎 | | | | 🞎 | | | | | | | | 🞎 | | | | | | 🞎 |
| **International Physical Activity Questionnaire (IPAQ)** | | | | | | | | | | | | | | | | | | | | | | |
| Think about all the ***vigorous activities*** that you did in the last 7 days. Vigorous physical activities refer to activities that take hard physical effort and make you breathe much harder than normal. Think only about those physical activities that you did for at least 10 minutes at a time. | | | | | | | | | | | | | | | | | | | | | | |
| 1. During the last 7 days, on how many days did you do vigorous physical activities like heavy lifting, digging, aerobics, or fast bicycling? | | | | | | | | | | | | 🞎 Yes  **…** days/week | | | | | | | | | 🞎 No  Skip to question 3 | |
| 1. How much time did you usually spend doing vigorous physical activities on one of those days? | | | | | |  | | | | | | | 🞎 Yes  **…**minutes/day | | | | | | | | 🞎 Not sure/Don’t know | |
| Think about all the ***moderate activities*** that you did in the last 7 days. Moderate activities refer to activities that take moderate physical effort and make you breathe somewhat harder than normal. Think only about those physical activities that you did for at least 10 minutes at a time. | | | | | | | | | | | | | | | | | | | | | | |
| 1. During the last 7 days, on how many days did you do moderate physical activities like carrying light loads, bicycling at a regular pace, or doubles tennis? Do not include walking. | | | | | |  | | | | | | | | 🞎 Yes  **…**days/week | | | | 🞎 No  Skip to question 5 | | | | |
| 1. How much time did you usually spend doing moderate physical activities on one of those days? | | | | | |  | | | | | | | | | 🞎 Yes  **…**minutes/day | | | | | 🞎 Not sure/Don’t know | | |
| Think about the time you spent ***walking*** in the last 7 days. This includes at work and at home, walking to travel from place to place, and any other walking that you have done solely for recreation, sport, exercise, or leisure. | | | | | | | | | | | | | | | | | | | | | | |
| 1. During the last 7 days, on how many days did you walk for at least 10 minutes at a time? | | | | | | | | |  | | | | | | 🞎 Yes  **…**days/week | | | | | | 🞎 No  Skip to question 7 | |
| 1. How much time did you usually spend walking on one of those days? | | | | | | | | | | 🞎 Yes  **…**minutes/day | | | | | | | | | | 🞎 Not sure/Don’t know | | |
| The last question is about the time you spent sitting on weekdays during the last 7 days. Include time spent at work, at home, while doing course work and during leisure time. This may include time spent sitting at a desk, visiting friends, reading, or sitting or lying down to watch television. | | | | | | | | | | | | | | | | | | | | | | |
| 1. During the last 7 days, how much time did you spend sitting on a week day? | | | | | | | 🞎 Yes  **…**minutes/day | | | | | | | | | | | | 🞎 Not sure/Don’t know | | | |
