# Supplementary material for: Adherence to Mediterranean diet, physical activity level, and severity of periodontitis: Results from a university‐based cross‐sectional study
Source: J Periodontol. 2022 Feb 25;93(8):1218–32. doi: 10.1002/JPER.21-0643 (PMC9544461; doi:10.1002/JPER.21-0643)
Supplement: Supplementary file 4 — Supplementary Table 2: Post‐hoc power analysis for the association between Stage III/IV periodontitis and each evaluated risk indicator. [file JPER-93-1218-s006.docx]

| **Risk indicators for SIII-IV periodontitis** | **Alfa** | **Sample Size** | **Power** |
| --- | --- | --- | --- |
| Low MD adherence | 0.05 | 235 | >90% |
| Low PA level | 0.05 | 235 | 76.66% |
| Low MD adherence and low PA level | 0.05 | 235 | >90% |

**Supplementary Table 2**: *post-hoc* power analysis for the association between stage III/IV periodontitis and each evaluated risk indicator.

*Note*. SIII-IV, Stage III/IV periodontitis; MD, Mediterranean Diet; PA, Physical Activity.
