# Supplementary material for: Adherence to Mediterranean diet, physical activity level, and severity of periodontitis: Results from a university‐based cross‐sectional study
Source: J Periodontol. 2022 Feb 25;93(8):1218–32. doi: 10.1002/JPER.21-0643 (PMC9544461; doi:10.1002/JPER.21-0643)
Supplement: Supplementary file 5 — Supplementary Table 3: Association between aMed and physical activity level with periodontitis case. [file JPER-93-1218-s004.docx]

| **Variable** | **ORs for Periodontitis case** | | | | | | | |
| --- | --- | --- | --- | --- | --- | --- | --- | --- |
|  | **Crude ORs** | **95% CI** | | ***p*-value^*^** | **Adjusted**^†^ **ORs** | **95% CI** | | ***p*-value^*^** |
|  |  | **Lower** | **Upper** |  |  | **Lower** | **Upper** |  |
| Low/Moderate PA level | 0.96 | 0.44 | 2.15 | 0.93 | 1.31 | 0.51 | 3.35 | 0.58 |
| Low aMed^‡^ | 1.24 | 1.01 | 2.03 | *0.04* | 1.57 | 1.11 | 2.48 | ***0.02*** |
| *aMed and PA level* | | | | | | | | |
| Low aMed, low/moderate PA | 1.38 | 1.02 | 4.58 | *0.03* | 1.53 | 1.04 | 5.74 | ***0.01*** |
| Low aMed, high PA | 0.99 | 0.25 | 1.44 | 0.07 | 1.26 | 0.61 | 2.69 | 0.09 |
| High aMed^‡^, low/moderate PA | 0.35 | 0.11 | 1.08 | 0.09 | 0.61 | 0.21 | 1.78 | 0.34 |
| High aMed, high PA | REF. |  |  |  |  |  |  |  |
| *aMed components* | | | | | | | | |
| Wholegrain products | 0.55 | 0.26 | 1.19 | 0.13 | 0.64 | 0.26 | 0.96 | ***0.00*** |
| Vegetables | 1.54 | 0.73 | 3.23 | 0.26 | 0.98 | 0.81 | 1.22 | 0.12 |
| Fruits | 1.24 | 0.59 | 2.58 | 0.56 | 1.00 | 0.78 | 1.26 | 0.45 |
| Olive oil | 0.32 | 0.11 | 0.75 | *0.02* | 0.29 | 0.09 | 0.77 | ***0.01*** |
| Wine | 0.59 | 0.19 | 1.78 | 0.35 | 0.61 | 0.12 | 1.67 | 0.49 |
| Red meat and meat products | 1.04 | 0.48 | 2.27 | 0.51 | 1.44 | 0.59 | 3.47 | 0.65 |
| Fish | 0.74 | 0.36 | 1.54 | 0.41 | 0.83 | 0.35 | 1.94 | 0.66 |
| Dried fruits | 0.97 | 0.47 | 2.01 | 0.94 | 0.95 | 0.39 | 2.31 | 0.91 |
| Pulses | 1.09 | 0.50 | 2.37 | 0.82 | 1.10 | 0.47 | 2.54 | 0.82 |

**Supplementary Table 3**: Association between aMed and physical activity level with periodontitis case.

Abbreviations: ORs, odds ratios; CI, confidence interval; PA, physical activity; aMed, alternate Mediterranean diet score; REF., reference category.

**^*^** *p*<0.05

^†^Adjusted for age, gender, smoking and brushing frequency.

^‡^ High aMed if aMed>4; Low aMed if aMed<5.
