# Supplementary material for: Adherence to Mediterranean diet, physical activity level, and severity of periodontitis: Results from a university‐based cross‐sectional study
Source: J Periodontol. 2022 Feb 25;93(8):1218–32. doi: 10.1002/JPER.21-0643 (PMC9544461; doi:10.1002/JPER.21-0643)
Supplement: Supplementary file 6 — Supplementary Table 4: Results of the mediation analysis of BMI on the effect of the combination of diet and physical activity on the presence of Stage III/IV periodontitis. [file JPER-93-1218-s005.docx]

| **Effect** | **Coefficient** | **SE** | **z** | ***p*-value** |
| --- | --- | --- | --- | --- |
| BMI <- combined aMed/PA (Step 1) | 0.531 | 0.262 | 2.03 | *.042* |
| SIII-IV <- BMI (Step 2) | 0.027 | 0.006 | 4.56 | *.000* |
| SIII-IV <- combined aMed/PA (Step 3) | 0.156 | 0.024 | 6.41 | *.000* |
|  | **Results of the Sobel test for the indirect effect** | | | |
|  | **Coefficient** | **SE** | **z** | ***p*-value** |
|  | 0.015 | 0.008 | 1.854 | .064 |
|  | *Indirect effect/Total effect= .085* | | | |

**Supplementary Table 4**: results of the mediation analysis of BMI on the effect of the combination of diet and physical activity on the presence of Stage III/IV periodontitis.

*Note*. SE, Standard Error; BMI, Body Mass Index; aMed, adherence to Mediterranean Diet; PA, Physical Activity; SIII-IV, Stage III-IV periodontitis.
